# Vaccination Failure in Eradication and Control Programs for Bovine Viral Diarrhea Infection

**DOI:** 10.3389/fvets.2021.688911

**Published:** 2021-06-29

**Authors:** Aleksandra Antos, Pawel Miroslaw, Jerzy Rola, Miroslaw Pawel Polak

**Affiliations:** Department of Virology, National Veterinary Research Institute, Pulawy, Poland

**Keywords:** bovine viral diarrhea, BVDV, vaccination, control, genetic diversity, cross neutralization

## Abstract

Vaccination against bovine viral diarrhea (BVD) is one of the key elements to protect cattle herds from this economically important disorder. Bovine viral diarrhea virus (BVDV) is a pestivirus infecting animals at all ages with significant impact on reproductive, digestive, and respiratory systems. Financial burden caused by this pathogen prompts many farmers to introduce vaccination as the control and prophylactic measure especially when persistently infected (PI) individuals, being the main source of the virus in the herd, are removed after test-and-cull approach. The aim of the study was to compare the serological response in cattle herds where new PI calves were identified without prior removal of PI animals or despite their removal and after the introduction of whole herd vaccination against BVDV infection. Overall seroprevalence in 5 vaccinated herds was 91.7 and 83.3% using ELISA and virus neutralization test, respectively. Despite high titers for both vaccine and field strains of BVDV in analyzed herds the analysis of comparative strength of neutralization indicated that 41.4% of positive samples did not have a predominant titer against one specific subtype of BVDV. In 3 herds BVDV-1b subtype was identified while in 2 others it was BVDV-1d, while the vaccine used was based on BVDV-1a which was never identified in Poland so far. To increase the success of the BVDV eradication program, a careful approach is suggested when planning herd vaccination. Comparison of existing field strains and their similarity with vaccine strains at antigenic and genetic levels can be a useful approach to increase the effectiveness of vaccination and efficient protection of fetuses from persistent infection.

## Introduction

Bovine viral diarrhea (BVD) is one of the most important infectious viral diseases of cattle, caused by bovine viral diarrhea virus (BVDV), with an enormous economic and animal welfare impact on beef and dairy industries. This pathogen has a worldwide distribution and infects livestock and wildlife ruminants. BVDV belongs to the growing *Pestivirus* genus, within the family *Flaviviridae*. Based on the latest classification of the International Committee on Taxonomy of Viruses, genus *Pestivirus* is composed of 11 recognized species with 2 species of BVDV, namely *Pestivirus A* (according to former nomenclature: Bovine viral diarrhea virus species 1 – BVDV-1) and *Pestivirus B* (Bovine viral diarrhea virus species 2 – BVDV-2). Molecular typing allowed distinction of at least 23 subtypes within BVDV-1 and 4 within BVDV-2 (1, 2). Additionally, both virus species occur as two biotypes, i.e., cytopathic (cp) and non-cytopathic (ncp), according to their ability to induce cell damage in cell culture (3). The single positive-stranded RNA of BVDV genome contains a single large open reading frame encoding a polyprotein that is cleaved by viral and cellular proteases into structural (C, E^rns^, E1, E2) and non-structural (N^pro^, NS2-3, NS4A, NS4B, NS5A, NS5B) proteins. From all viral proteins the non-structural protein NS3 and the glycoproteins E^rns^ and E2 are the immunodominant proteins of BVDV, which induce significant and detectable antibody titers in infected animals (4, 5).

Infections with BVDV cause a wide range of clinical symptoms, from mild clinical signs to severe form terminated by death, depending on the virulence of the strain, reproductive and immune status of the animal, and its age (6). A severe clinical form of BVD known as mucosal disease (MD) is 100% fatal. Mucosal disease occurs only in cattle persistently infected with BVDV when they become infected with a cytopathic strain of BVDV, homologous to persisting strain. It may be the result of a natural infection or post-vaccinal reaction, which occurs after vaccination with a modified live virus (MLV) BVDV vaccine. This phenomenon applies only to vaccines that contain the cp biotype of the virus, but it happens very rarely (7). BVDV can spread horizontally, usually by direct contact with other infected animals, causing transient infection (TI) that lasts 2–3 weeks before the animal becomes immune and high levels of antibodies can persist even for the rest of the animal's life. Vertical transfer of the virus during pregnancy may result in fetal infection, which can lead to abortions, teratogenic effects, or the birth of persistently infected (PI) and immunotolerant calves (8). PI animals play an important role in any control or eradication program. PIs shed virus in high concentrations throughout their lives and they are a main reservoir of infection in the herd (9, 10).

BVDV-1 is the most widespread ruminant pestivirus worldwide, whereas subtypes 1a and 1b are the most common and the most studied ones (1). Epidemiological data from Poland indicates that BVD infection is ubiquitous, and more than 70% of dairy herds have been found to be seropositive when bulk tank milk was tested (11). A similar study conducted by Rypuła et al. (12) showed a high percentage of BVDV-positive animals, especially in large dairy herds. The most predominant subtypes of BVDV detected in Poland were BVDV-1b and 1d (13), but subsequent studies indicated that over time besides BVDV-1b, also BVDV-1g and BVDV-1f subtypes are often identified (14). Another species, namely BVDV-2, has been identified in Poland but only in one vaccinated herd (15).

Due to the significant economic impact of BVD on cattle production, many countries including Norway, Sweden, Denmark, Finland, Austria, Switzerland, Germany, Ireland, Scotland, England, Wales, Belgium, the Netherlands, and the USA have implemented compulsory or voluntary control and/or eradication programs. Following these countries, Poland introduced a voluntary BVDV eradication program in early 2018. The first and fundamental principle of the successful BVD program is strict biosecurity with reliable diagnostics, followed by the elimination of PI animals from the herd. Next step is the prevention of the generation of new PIs, and stopping or limiting the transmission from infected individuals to susceptible animals. In addition to biosecurity, there should be an effective vaccination program designed to protect against BVDV, since it is a relatively inexpensive and effective tool. Prior to vaccination, PI animals should be identified and removed, as we have shown earlier that by omitting this step, it was not possible to protect the herd from new infections (15).

Modified live (MLV) and killed (KV) vaccines have been available for more than 50 years. The occurrence of BVD still remains a significant problem, implying that the vaccines need to be improved. The deficient effectiveness of BVDV vaccines is likely due to the huge heterogeneity among different viral strains most likely caused by the lack of proofreading activity of RNA polymerase during replication of viral genome and the resulting antigenic variability. It is desirable to achieve maximal response to vaccination at a minimal expense to avoid reduced performance (6, 16). Although the presence of neutralizing antibodies is frequently used as a measure of the immune response to vaccination, the titer of those antibodies required for protection against BVDV infection is still under discussion (17). Some authors indicate that 1/16 dilution is enough (18) while others refer to 1/128 (19), 1/256 (20), or even 1/512 (21) as protective dilution against BVDV-1. Additionally, cell-mediated immunity seems to play a crucial role in protective immunity since animals with low levels of antibodies were protected from viral challenge (22).

Vaccination in Poland relies on several vaccines containing mostly BVDV-1 (both MLV and KV) and only one MLV vaccine is available, which is composed of both BVDV-1 and BVDV-2 species. It was introduced in Poland 1 year after the first identification of BVDV-2 infection in native cattle (15). Currently, on the Polish market, there are four killed (inactivated virus) vaccines, one live attenuated, and one modified live vaccine (MLV). Three of them are multivalent with immunogens for BVDV along with parainfluenza 3 virus (PI3V), bovine respiratory syncytial virus (BRSV), and *Mannheimia haemolytica* immunogens. The major objective of BVDV vaccination is the prevention of transplacental infection of fetuses and thus stopping the birth of PI animals. Furthermore, an efficient vaccine should mediate cross protection against the circulating subtypes of BVDV-1 and BVDV-2. Although there have been multiple studies showing efficacy of BVDV-1a vaccine against BVDV-1b (23–25), other studies demonstrated lower antibody titers against different pestivirus species (17, 20, 26) and differences in antibody titers among various viral subtypes (27). Considering the increased genetic diversity of BVDV subtypes identified in Poland, a better understanding of the relationship between antigenic differences of BVDV is critical for the improvement of future vaccines.

The aim of this study was to assess the host response of vaccinated animals in herds where PI individuals were born despite the vaccination and to determine whether the emergence of a new virus subtype in a herd will influence antibody response.

The sequential aim was to assess differences in BVDV vaccine strains vs. PI field strains by molecular typing within 5'UTR and N^pro^ coding region.

## Materials and Methods

### Animals Tested and Vaccine Used

Five dairy herds (Table 1) were included in the study. In three of them (A, K, and L) vaccinations of whole herds were introduced after PI animals identification and removal and vaccination lasted for 1, 3, and 5 years, respectively. In all five herds calves and heifers were kept in the same buildings but in separate pens. In all herds the same KV vaccine containing BVDV-1a strain was used. All mothers of PI calves were vaccinated before insemination according to manufacturer's instructions. Primary vaccination in those herds was performed in 8 months old animals with booster 4 weeks later. Revaccinations were done every 6 months. Vaccine manufacturer claims that fetal protection is provided when second vaccine dose is given to a heifer or a cow to be inseminated 4 weeks before the start of gestation. Herd A was the only herd where the first vaccination and booster 4 weeks later were done and PIs in that herd were identified before annual revaccination.

**Table 1:** Detailed information on animals included in the study.

**Herd ID**	**Herd size**	**Number of samples tested**	**Age of animals tested in months**	**Duration of vaccination in years before testing**	**PI removal before vaccination**	**Clinical signs in vaccinated animals**
**A**	250	30	12	1	Yes	No
**OS1**	409	14	12	6	No	No
**OS2**	466	19	12	6	No	No
**K**	300	K1=20	4	3	Yes	Yes
		K2=20	12			
**L**	1,200	30	12	5	Yes	No

Respiratory symptoms and reproductive problems such as embryo resorption were observed only in herd K.

### BVDV Antibody Detection by ELISA

The presence of BVDV antibodies in bovine sera was tested with a commercial enzyme-linked immunosorbent assay (ELISA) kit. The test is based on the pestivirus envelope protein E^rns^ (BVDV Total Ab Test, IDEXX, Liebefeld-Bern, Switzerland) and it was used according to the manufacturer's instructions. This ELISA provides specificity and sensitivity of 97.1 and 96.7%, respectively, compared with the virus neutralization test (VNT) (28).

### BVDV Antibody Detection by Virus Neutralization Test (VNT)

Two-fold serial dilutions (from 1:5 up to 1:640) of serum samples inactivated at 56°C for 30 min and positive in antibody ELISA were tested for neutralizing antibodies against cytopathic (cp) BVDV-1a strain Singer and two non-cytopathic (ncp) field strains BVDV-1b (60-GB/11), BVDV-1d (142-GB/15), and BVDV-2a (CS8644). Madin-Darby bovine kidney (MDBK) cells supplemented with 10% fetal calf serum were used for VNT. Both cell culture and calf serum were free of BVDV and antibodies against this virus. Two wells per dilution of each sample were used. Fifty μL of BVDV-1 strains prepared in order to obtain 100TCID50 were added to duplicate wells. After 1 h of incubation at 37°C with 5% CO_2_, 100 μL of MDBK were added at a density of 150,000/mL. Plates were incubated for 4 days at 37°C in the incubator with 5% CO_2_. After incubation, the cells were observed for cytopathic effect in the case of Singer strain while ncp biotype was detected by indirect immunoperoxidase (IPX) method with primary monoclonal antibody WB103/105 (VLA Weybridge, UK) against Pestiviruses (Group specific). DAB substrate (SIGMA-ALDRICH, USA) was added to visualize infected cells. The antibody titers were determined as the reciprocal of the highest serum dilution, which neutralized the virus in at least 50% of the wells. Positive and negative control sera were included in each test. The calculated VN titers and the distribution of the data were represented by box and whisker plots. Additionally titers against different subtypes were calculated for specific ranges and presented as percentages. Negative samples were defined as negative in VNT or with titers up to 10, low titers samples were between 10 and 20, medium titers were 40–80, and high titers were 160–640. In herds K1, K2, and L the titers for heterologous strains (BVDV-1b and BVDV-2a) were also examined.

### BVDV Antigen Detection

A commercial ELISA which detects Pestivirus A, B, and H, based on the BVDV E^rns^ antigen (BVDV Ag/Serum Plus, IDEXX, Liebefeld-Bern, Switzerland) was used. Serum samples were tested according to the manufacturer's protocol. This test demonstrates specificity of more than 99.7% and a sensitivity of nearly 100% (29–31).

### Virus Detection

RNA was extracted from serum samples using TRI Reagent (SIGMA-ALDRICH, USA), according to the manufacturer's instructions. The RNA was eluted in diethylpyrocarbonate-treated water (Invitrogen, Carlsbad, CA, USA).

One-step standard RT-PCR was performed using a Transcriptor One-Step RT-PCR Kit (Roche Diagnostics GmbH) according to the manufacturer's instructions using the primers pair (324F-5'-ATG CCC WTA GTA GGA CTA GCA-3'; 326R-5'-TCA ACT CCA TGT GCC ATG TAC-3'), designed to amplify a 288-bp length fragment from the 5′UTR region of Pestivirus genome (32). Additional primers pair (B32-5′-TGC TAC TAA AAA TCT CTG CTGT-3′; B31-5′-CCA TCT ATR CAY ACA TAR ATG TGGT-3′) was designed to amplify a 441-bp length fragment from the N^pro^ region of BVDV (33). The final volume of RT-PCR reaction mixture was 25 μl including 23 μl of reaction mix and 2 μl of RNA template. The amplification of 5′UTR region was done at 50°C for 30 min and 94°C for 7 min, followed by 10 cycles of 94°C for 10 s, 53°C for 30 s, 68°C for 30 s, then 25 cycles of 94°C for 10 s, 53°C for 30 s, 68°C for 33 s with a final extension step at 68°C for 7 min, while the thermal profile for N^pro^ region was similar except for the annealing temperature decreased to 50°C.

The RT-PCR products were submitted to electrophoresis in 1.5% agarose gel in TBE buffer, stained with ethidium bromide, and visualized under UV light.

### Sequencing and Phylogenetic Analysis

The products of the standard RT-PCR were purified and sequenced as described previously (13). Phylogenetic analysis was done by the neighbor-joining (NJ) statistical method with the Kimura two-parameter model using MEGA software (version 5.03). The reliability of the constructed phylogenetic trees was evaluated by running 1,000 bootstrap replications in the phylogeny test and bootstrap values ≥70% were considered good support.

### Data Analysis

To compare the predominance of titers against the different BVDV subtypes, a formula established by Silveira et al. (34) was used for determining the comparative ratio (R) for each serum sample: R_subtypeA_ = (4 × titer against subtype A)/(titer against subtype B + titer against subtype C + titer against subtype D). If the value for one subtype was >0.231 than the value for the other subtype, the sample was considered to have predominant titer for the respective subtype. If the ratio value for all subtypes was <0.231 among them, the sample was considered to be without a predominant titer.

The Shapiro-Wilk test calculation for normality and box and whiskers plots were made using R 4.0.4 for Windows Software, which is an open source project that is distributed under the GNU General Public License.

### Ethics

Samples and data were collected as a part of routine clinical examination of the animals and this survey did not involve experimental studies. Samples were collected from animals by local vets after verbal approvals from the owners for further testing. No extra animal discomfort was caused for sample collection for the purpose of this study. The approval from ethics committee was not required according to national regulation (“Act on the Protection of Animals Used for Scientific or Educational Purposes” published in the *Journal of Laws* of 2015, item 266 from 15 January, 2015).

## Results

### Antibody Detection

The number of positive, doubtful, and negative samples among the 133 sera tested by antibody ELISA was 122, 3, and 8, respectively. All doubtful and six negative results were obtained in herd K1, while other two negative results were identified in herd K2. The S/P values for positive samples were very high. All serum samples were tested also by VNT and 83.3% of them were positive (a titer of 10 and above), for at least one BVDV subtype used.

**Figure 1:**
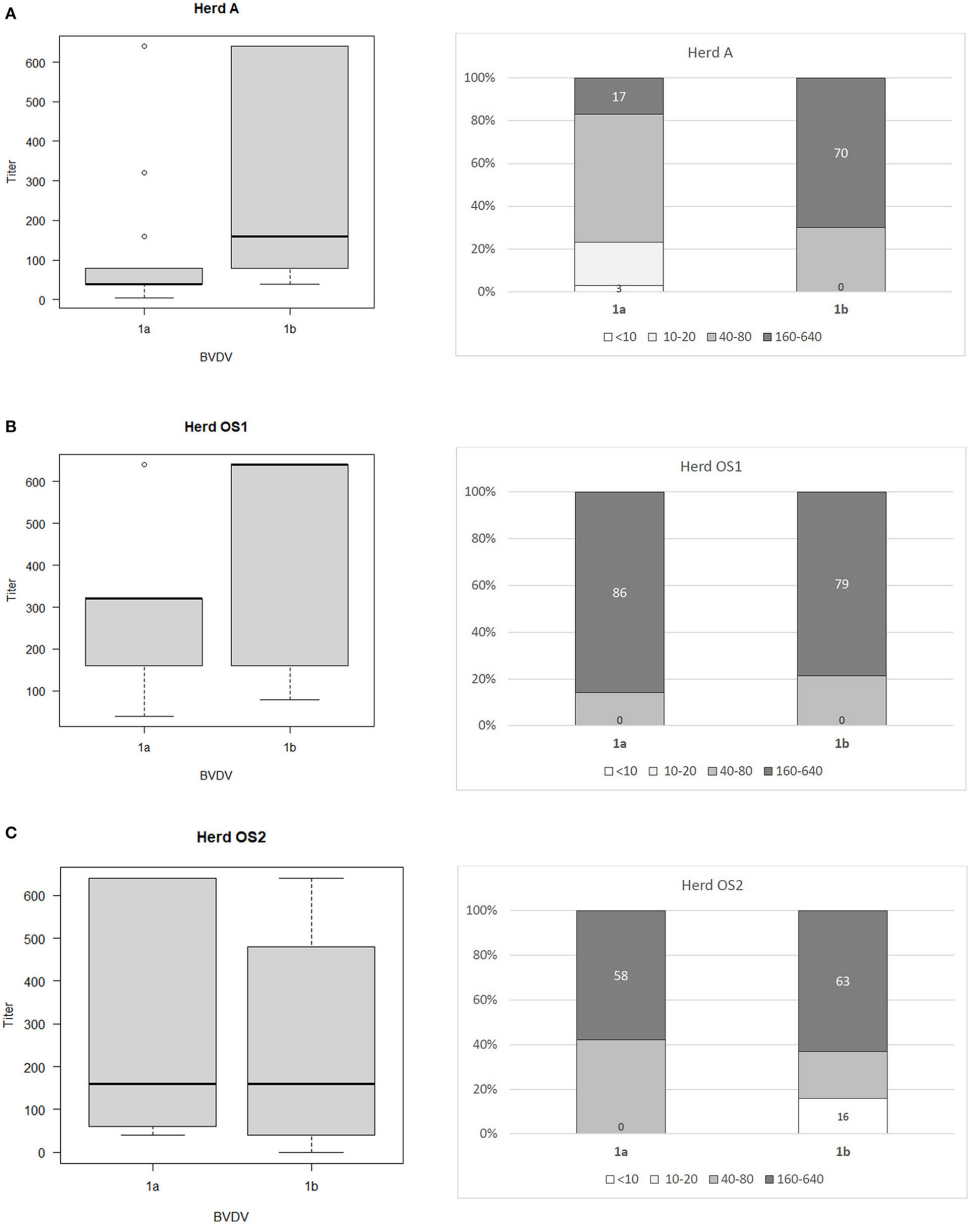
Box and whiskers plots of antibody titers juxtaposed with percentages of antibody titers against BVDV-1a and BVDV-1b for herds where PI animals infected with BVDV-1b were identified: **(A)** herd A, **(B)** herd OS1, **(C)** herd OS2. The top and bottom of boxes represent 25th and 75th percentiles, respectively; the middle line represents the median value, whiskers represent the highest and lowest values which are not outliers, outliers are indicated as circles. In a percentage graph, samples were classified as negative (VN titers up to 10), low (titers between 10 and 20), medium (titers between 40 and 80), and high (titers between 160 and 640) titer samples. Numbers refer to percentages of negative and high titer samples.

**Figure 2:**
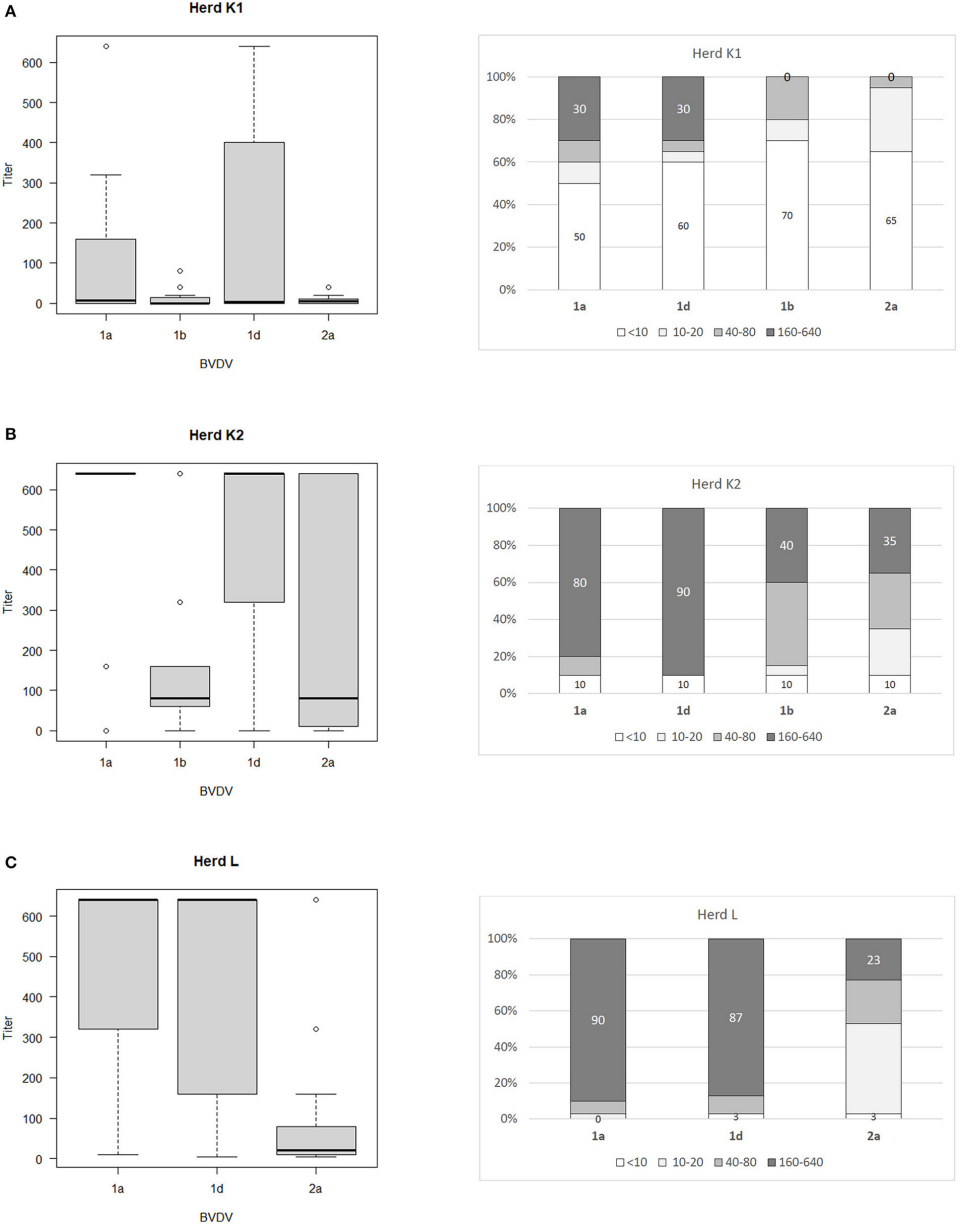
Box and whiskers plots of antibody titers juxtaposed with percentages of antibody titers against BVDV-1a, BVDV-1b. BVDV-1d, and BVDV-2a for herds where PI animals infected with BVDV-1d were identified: **(A)** herd K1, **(B)** herd K2, **(C)** herd L. The top and bottom of boxes represent 25th and 75th percentiles, respectively; the middle line represents the median value, whiskers represent the highest and lowest values which are not outliers, outliers are indicated as circles. In a percentage graph, samples were classified as negative (VN titers up to 10), low (titers between 10 and 20), medium (titers between 40 and 80), and high (titers between 160 and 640) titer samples. Numbers refer to percentages of negative and high titer samples.

The ranges of antibody titers for specific subtypes of BVDV are presented in Figure 1 (where BVDV-1b was identified) and Figure 2 (where BVDV-1d was detected) with median values, highest-lowest values, and percentages of antibody titers for vaccine and field strains in respective herds separated into 4 groups as negative, low, medium, and high positive samples. High titers (80–90% of all titers) for vaccine strain were predominant in herds K, L, and OS1 while in herd A it was only 17%. In case of field strains high titers for homologous subtypes were between 63 and 90%. Surprisingly in two herds with BVDV-1d high titers against BVDV-2a were identified in 23 and 35% of all samples with positive titers. Level of high titers in 4 months old calves from herd K1 was the lowest reaching only 30% for both vaccine and field strain of BVDV.

To determine the comparative strength of neutralization we adopted the formula established by Silveira et al. (34). Within the positive samples, 41.4% did not have a predominant titer against one specific subtype of BVDV. Only in 16.5% of positive samples BVDV-1a (vaccine strain) predominated and for 31.6%, the titer against the BVDV subtype detected in PI animals was the dominant one. Regarding 10.5% of the remaining sera, they showed predominant titers for a subtype of BVDV different from vaccine and PI strains. Within this last category 12 samples (17.1%) had predominant titers for the BVDV-2a subtype.

### Detection of PI Animals by RT-PCR and Antigen ELISA

PI animals were identified in one 4-months-old heifer in herd A, two 4-months-old heifers in herd K1, one 8-months-old heifer in herd K2, one 4-months-old heifer in herd L, five calves and heifers 1–6 months old in herd OS1, and in seven heifers 3–9 months old in herd OS2. PI status of all animals positive in the first test (RT-PCR with 5′UTR primers) was confirmed by second positive test result after 3–4 weeks from the first test with antigen ELISA (BVDV Ag/Serum Plus, IDEXX, Liebefeld-Bern, Switzerland). The source of infection in those herds was not identified except herd K2, where 60 heifers were purchased from outside, without testing for BVDV, before the time when PIs could be generated. Soon after that respiratory signs in calves and embryonic deaths in pregnant females were recorded. None of the PI calves developed clinical signs of MD.

### Phylogenetic Analysis

Standard RT-PCRs targeting two regions of the BVDV, namely 5′UTR and N^pro^, were used. Both genome regions are the most frequently used in the molecular characterization of pestiviruses. 5′UTR sequences were obtained from a total of 6 BVDV-1 positive samples. For 4 of them, the sequence of the N^pro^ region was also generated. BLAST search and analysis with reference strains from GenBank showed that identified isolates belonged to BVDV-1b (herds A, OS1, and OS2) and BVDV-1d (herds K1, K2, and L).

**Figure 3:**
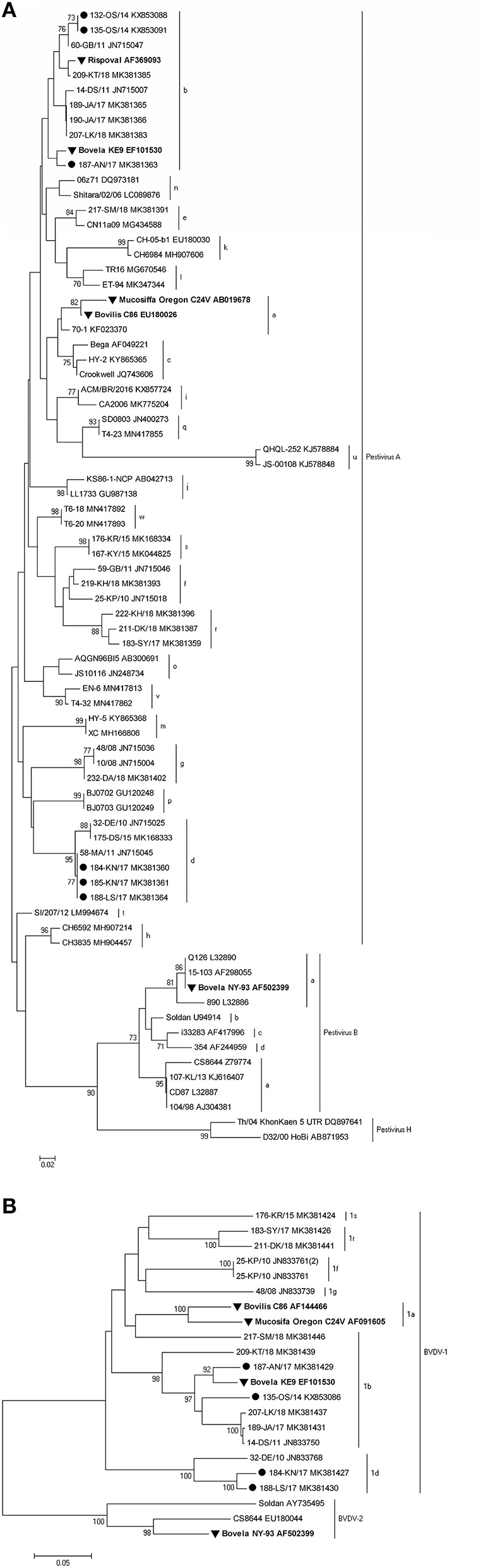
Phylogenetic trees based on the partial **(A)** 5′UTR and **(B)** N^pro^ sequences obtained from vaccinated herds. Strains reported in this study are marked with a black circle, and vaccine strains are labeled in bold and marked with a black triangle. The numbers close to the major nodes indicate the bootstrap values as percentage. Reference sequences were retrieved from GenBank and the accession numbers are given after each strain.

A neighbor-joining tree was constructed which confirmed the subtyping obtained by sequence analysis, clustering the strains inter alia with the same subtypes detected earlier in Poland. To confirm the grouping within the 5′UTR region, sequences of the partial N^pro^ region of 4 viruses were analyzed. Representative strains from all farms are presented in Figure 3A for the 5′UTR region and in Figure 3B for the N^pro^ region, both along with vaccine strains available in the GenBank and subtype specific strains from earlier studies in Poland (identified by 2–3 digits and followed by two letters identifying the herd of origin). The GenBank accession numbers of sequences of virus strains used in phylogenetic analyses are shown in the figures.

The nucleotide identity, calculated with BioEdit (version 7.2.5), for BVDV-1b and BVDV-1d strains detected in this survey was in the range of 99.6–100 and 99.2%, respectively. Such a high similarity of the analyzed sequences may indicate one strain introduction into the herd.

## Discussion

Our study identified 5 herds where PI animals were detected despite ongoing vaccination against BVD. Field strains from PI individuals were of different subtypes from vaccine strain of BVDV. In three herds (marked as K, A, and L) vaccination followed the identification and removal of PI animals. In remaining 2 herds (OS1 and OS2) PIs were not identified and removed before the vaccination. The owners of those two herds expected that natural pressure from vaccine strain of BVDV will allow to get rid of virus source in a longer run so the vaccination was continued for 6 years before testing the whole herd for persistently infected animals. Despite different strategies, in both types of herds the vaccine did not protect the fetuses from intrauterine infection with BVDV subtypes different from the vaccine strain leading to the birth of virus shedders.

Extensive genetic variability of different strains of BVDV-1 (23 subtypes) and BVDV-2 (4 subtypes) hampers the success of vaccination worldwide. According to VIOLIN database (35), currently almost 130 licensed vaccines for BVD are available commercially and despite their common use many herds are not free from the virus and reinfections occur frequently.

In two retrospective phylogenetic studies of BVDV positive samples collected in Poland in years 2004–2011 and 2015–2018, which were based on 5′-untranslated region (5′-UTR) and N^pro^ coding sequences, 4 and 7 subtypes of BVDV were identified, respectively, but not BVDV-1a (13, 14). In the latter study predominant subtypes were BVDV-1b, BVDV-1g (27% each of all subtypes identified), and BVDV-1f (24%). BVDV-1d, which was second predominant subtype in Poland in years 2004–2011 (37% compared to 48% of BVDV-1b) was identified in 9% of all positive samples detected in 2015–2018. In this study two subtypes of BVDV were identified in 5 vaccinated herds: BVDV-1b in three herds and BVDV-1d in two. When evaluating the efficacy of vaccines based on BVDV subtypes different from field isolates one has to bear in mind that subtype classification is usually based on sequence identity in regions of viral genome (like 5′UTR or Npro) not related to coding regions of viral immunogenic proteins like E2 or NS2-3. Therefore, significant antigenic differences are observed even within the same subtypes like in BVDV-1b strains from Argentina (36). While several years ago only 2 vaccines against infection with BVDV were available on Polish market now we have access to 6 biologicals (2 of them are modified live virus vaccines while 4 are killed vaccines). In all 5 herds described in this study the same inactivated vaccine based on BVDV-1a was used. Vaccination was done for six consecutive years in herds OS1 and OS2, for 5 years in herd L, 3 years in herd K, and for 1 year only in herd A (first vaccination and booster 4 weeks later).

Earlier, BVDV-2a was detected in one Polish herd despite the introduction of vaccination with another killed vaccine containing BVDV-1a after the appearance of respiratory signs in calves and heifers (15). Additionally, deaths of newborn calves with bloody diarrhea were recorded. Despite implemented vaccination transient infection with BVDV-2 was confirmed in 7 heifers. Four PI animals were identified 1 year after the vaccination started although two of them were 1 year old heifers so the virus could be present in that herd earlier. Similar outcome was observed in the study of viruses isolated from PI calves born to dams vaccinated against BVDV before breeding (37). The genotype of BVDV most often isolated from such animals (BVDV-2) was different from the vaccine virus (BVDV-1). However, in that study MLV vaccine was used and the study was done in the region where BVDV-2a was a predominant species of the virus. Similar outcome was described in beef herds which were also vaccinated with a modified-live BVDV-1 vaccine (38).

In another study evaluating vaccine efficacy, BVDV-1b infected PI calves were introduced to a herd consisting of calves coming from two sources and vaccinated against BVDV in their farms of origin with a killed and a modified live vaccine both containing BVDV-1a and BVDV-2a strains (39). Titers against BVDV-1b up to 64 did not prevent viremia while titers up to 256 did not prevent 4-fold increases in BVDV-1b antibody titers confirming seroconversion. Therefore, even when antibodies to BVDV strain shed by a PI individual were pre-existing before challenge but at low titer they could not protect all animals against the infection. In our study VN titers against both vaccine and field strains of BVDV in tested herds were quite widespread from low (below 20) to high levels (up to 2,560 and 5,120). Although clinical signs were observed only in one herd, all vaccinated herds experienced the birth of PI calves, proving lack of fetal protection from the vaccine. The majority of those herds were closed units without purchase of animals from outside but the introduction of replacement heifers took place in herd K, where soon after that several abortions and embryonic deaths were identified. Much more diverse situation with respect to subtypes identified was described in a regularly vaccinated Brazilian herd (40). Animals in that herd were vaccinated twice a year with a commercial inactivated and multivalent vaccine containing BVDV-1a. Four PI animals were identified and they were infected with three different BVDV subtypes: BVDV-1a, BVDV-1b, and BVDV-1d. Such a diversity of BVDV subtypes in one herd could be related to the open cattle management system used to raise the animals in that herd with constant introductions of new animals from external sources. Despite regular vaccinations in this herd repeated breeding and increased embryonic deaths were diagnosed.

Rodning et al. (24) compared 3 commercial vaccines for preventing PI generation, including one inactivated vaccine containing BVDV-1a and BVDV-2. Heifers were bred by artificial insemination and had contact with PI calves between 68 and 126 days of pregnancy. PI calves were only produced in control group and in 2 out of 18 calves born from heifers vaccinated with inactivated vaccine. These two PI calves were infected with BVDV-1b and BVDV-2. Full protection against the development of PI calves was provided by 4 vaccinations with modified-live vaccine between weaning and breeding. On the other hand, 4 vaccinations with inactivated vaccine given also between weaning and breeding provided 89% protection. But, when inactivated vaccine was given according to manufacturer's instructions (2 doses instead of 4), protection from PI generation was only 73% (41). Full protection against the birth of PI calves after vaccination with inactivated vaccine was achieved only when vaccine and field strains were of the same subtype (BVDV-1a) (42). Some of the vet practitioners in the field also vaccinate cattle with higher number of doses than advised. In one herd a live vaccine was used every 6 months (like inactivated vaccine) opposite to manufacturer's advice to vaccinate every 12 months (personal communication). The results of this approach were satisfactory enough for the farmer to accept the higher cost of vaccination.

Sozzi et al. (43) analyzed cross reactivity antibody response after vaccination to other viral subtypes than those contained in vaccines used. One inactivated and three modified live vaccines were used harboring subtypes BVDV-1a and BVDV-1b. Cross reactive response was assessed for two strains of subtypes BVDV-1a and BVDV-1b each and one isolate of BVDV-1e. Only two modified live vaccines were able to induce detectable levels of cross reacting antibodies against at least one other subtype. The authors confirmed previous observations (7) of low level antibody response to BVDV-1b by BVDV-1a based vaccines. In our study of 3 herds where BVDV-1b PIs were detected, percentage of high VN titers (160 and above) against BVDV-1a and BVDV-1b was similar in herds OS1 (86/79%) and OS2 (58/63%) while in herd A, where vaccination was done only for 1 year 17% of VN titers against BVDV-1a and 70% of VN titers against BVDV-1b were high. Prevalence of PI animals in herds OS1, OS2, and A was 1.15, 1.5, and 0.4%, respectively. In herds K and L, where BVDV-1d was identified prevalence of PI animals was 1 and 0.1%, respectively.

Another approach was proposed by Mosena et al. (44). They used a multivariate analysis to assess the antigenic relationship between vaccine strains and field isolates of BVDV. VNT results were interpreted using principal component analysis (PCA) to get clustering patterns. Using this approach they identified single BVDV-1a and BVDV-2a strains which did not cluster anti-genetically with genetically similar subtypes. Such an approach provides a useful tool to better understand antigenic relationships between different isolates of BVDV even when they belong to the same subtype, which can improve future vaccine efficacy.

Newcomer et al. (45) in a meta-analysis of previously published studies tried to evaluate the efficacy of BVDV vaccination in preventing reproductive losses like risk of fetal infection, risk of abortion, and pregnancy risk. Overall it was concluded that vaccination with any type of vaccine (modified-live or inactivated, monovalent or polyvalent) provided significant protection against reproductive disease. Fetal infection could be decreased by 85%, abortion risk by nearly 45%, and only pregnancy risk was increased by 5% when compared with unvaccinated controls.

When analyzing vaccine failures one has to remember that some major assumptions have to be met but usually cannot be verified before blaming a given vaccine for the lack of immune protection against different viral subtypes. Vaccine has to be handled properly before and during vaccination (especially modified live vaccines), all eligible animals should be vaccinated, appropriate protective immunity should be generated in all vaccinated individuals, and future revaccinations should be continued according to vaccine manufacturer's recommendations (37).

In summary, the level of antibody titers against vaccine strain of BVDV was dependent on the duration of vaccination. Despite high titers for both vaccine and field strains of BVDV in analyzed herds the analysis of comparative strength of neutralization indicated that 41.4% of positive samples did not have a predominant titer against one specific subtype of BVDV. Only in 16.5% of positive samples vaccine strain predominated while for 31.6%, the titer against the subtype detected in PI animals was the dominant one. The prevalence of PI animals was the highest (1.5%) in the herd with 6 years history of vaccination and 7 virus positive animals identified. Percentage of high titers for heterologous strains was much lower than for homologous strains (40% for BVDV-1b in a herd infected with BVDV-1d and 23/35% for BVDV-2a in two herds infected also with BVDV-1d). Titers in 4-month-old calves (colostrum immunity) were very low with 50 and 60% of negative samples for vaccine and field strain of BVDV, respectively. Titers with values between 160 and 640 in calves comprised only 30% for both vaccine and field strain of BVDV. When comparing sequence identity within 5′UTR region of vaccine and field strains of bovine pestiviruses, subtype BVDV-1d is located furthest from sequences of available vaccines which could influence vaccine efficacy.

Low number of analyzed herds and various numbers of subtypes tested in the herds from this study could influence the general conclusions of vaccine efficacy, and further studies are needed to clarify this issue.

## Data Availability Statement

The datasets presented in this study can be found in online repository. The name of the repository and accession number(s) can be found at: https://www.ncbi.nlm.nih.gov/genbank/, KX853088, KX853091, MK381363, MK381360, MK381361, MK381364, MK381429, KX853086, MK381427, MK381430.

## Author Contributions

AA and MPP contributed initial conceptualization and design of the study, investigation, collection of the data, and writing—original draft, review and editing of entire manuscript. PM contributed investigation, collection of the data, revision, and read and approved the manuscript. JR contributed to manuscript revision and read and approved the submitted version. All authors contributed to the article and approved the submitted version.

## Conflict of Interest

The authors declare that the research was conducted in the absence of any commercial or financial relationships that could be construed as a potential conflict of interest.
